# The Effect of Hydrogen Peroxide on Sarco/Endoplasmic and Plasma Membrane Calcium ATPase Gene Expression in Cultured Human Lens Epithelial Cells

**DOI:** 10.2174/1874364100802010123

**Published:** 2008-07-07

**Authors:** M.J Marian, P Mukhopadhyay, D Borchman, D Tang, C.A Paterson

**Affiliations:** 1 Department of Biochemistry and Molecular Biology, University of Louisville School of Medicine, Louisville, KY 40202, USA; 2 Department of Molecular, Cellular and Craniofacial Biology, University of Louisville School of Dentistry, Louisville, KY 40202, USA; 3 Department of Ophthalmology and Visual Sciences, University of Louisville School of Medicine, Louisville, KY 40202, USA

## Abstract

The loss of calcium homeostasis in the lens of the eye appears to be a factor contributing to lens opacity. In the human lens, calcium homeostasis depends on the Ca^2+^-ATPase pumps found only in the epithelium. A plasma membrane calcium pump, PMCA2 is upregulated in human cataractous lenses. To determine if oxidation caused the plasma membrane Ca^2+^-ATPases (PMCA) or sarcoplasmic/endoplasmic Ca^2+^-ATPases (SERCA) to become upregulated, we cultured a human lens epithelial cell line, in the presence of hydrogen peroxide. We observed an increase in PMCA1, PMCA2 SERCA2b and SERCA3 mRNA levels and protein expression with increasing hydrogen peroxide concentrations and treatment times. Hydrogen peroxide caused a rise in the intracellular calcium which could be an initiating factor in the concerted upregulation of PMCA1 and SERCA3. Our data support the idea that oxidative stress could contribute to a selective rise in PMCA/SERCA expression in human cataractous lenses.

## INTRODUCTION

In the lens, cellular calcium homeostasis is attained by a delicate balance between passive inward movement from the extracellular milieu through membrane channels [1], extrusion by plasma membrane calcium ATPase (PMCA) [2], sodium calcium exchange [3], and internal sequestration by sarcoplasmic/endoplasmic reticular calcium ATPase (SERCA) [4]. There are equal amounts of the PMCA and SERCA proteins in the lens [5]. In the human lens, the Ca^2+^-ATPase pumps are found only in the epithelium [6-9], a single layer of cells on the anterior surface beneath the lens capsule. Human lens fiber cells contain few or no intracellular organelles and no Ca^2+^-ATPase [6,7,9]. It is important to define the role of this pump in the human lens, especially in light of the study showing that Ca^2+^-ATPase activity is 50% lower in human cataractous lenses [6].

Oxidation is a major factor in cataract development [10-15]. The lens Ca^2+^-ATPase pumps are very sensitive to oxidation [16-18] and oxidative inhibition of the lens Ca^2+^-ATPase can be reversed [18], however, inhibition of SERCA and PMCA may occur through a different mechanism [19,20]. Elevated intracellular calcium induces the upregulation of PMCA1 out of 4 PMCA isoforms [21], and both SERCA2 and SERCA3 [21,22] isoforms in an immortalized cell line of human lens epithelium. The oxidant hydrogen peroxide can lead to epithelial cell death and cataract [14,23-26]. Hydrogen peroxide levels are elevated in both the vitreous and lens of cataractous human lenses compared to clear lenses [27,28]. The expression of numerous proteins [29,30], including an increase in PMCA1 [31], are altered in lens epithelial cells treated with hydrogen peroxide. The expression of SERCA is carefully controlled and changes in SERCA expression may contribute to the etiology of many diseases including Brodie’s disease [32], Darier’s disease [33], and heart failure [34]. Oxidative stress reduces SERCA activity [35], however, it is not known if this reduction in activity is related to a decrease in SERCA protein or mRNA levels. In human cataractous lenses PMCA2 mRNA and protein levels are elevated compared to age matched clear lenses [36]. The purpose of this study was to determine if the expression of SERCA and PMCA isoforms are changed by hydrogen peroxide.

## MATERIALS AND METHODOLOGY

A human lens epithelial cell line (HLE B-3) was developed and provided by Andley *et al*. [37]. The human lens epithelial cells were immortalized by transfecting them with adenovirus 12-simian virus (Ad12-SV40) to maintain propagation of the cells *in vitro* [38]. Cell culture conditions, chemicals, membrane preparation, RNA extraction and Quantitative Real Time PCR (TagMan^®^, applied Biosystems, Foster City, CA), Electrophoresis and Western blotting and statistical analysis were performed exactly as described in citation [21].

### Hydrogen Peroxide Treatment

To study the effects of H_2_O_2_ as an oxidizing agent on the lens epithelial cells, at different exposure times and with different dosages, when cells were ~ 80% confluent, different concentrations of H_2_O_2_ ranging from 10 to 200 µM were added to the medium and cells were cultured for 4 hours. Untreated cells were used as a control. In a separate, but similar study, the cells at ~ 80% confluence were treated with 10 µM H_2_O_2_ for 4, 8, 16 hours. Untreated cells were used as a control. To inhibit catalase activity, 3-amino-triazole was added to the cell culture at 20 mM final concentration. The medium was replaced every 6 hours with fresh medium containing 10 µM H_2_O_2_ because H_2_O_2_ disappears from cell culture environment after 4 to 6 hours regardless of the presence of catalase inhibitor. Cells were analyzed microscopically with regard to their morphology and viability.

### Measurement of Intracellular Calcium

Intracellular ionized calcium concentration was measured using Indo-1 AM dye [39]. Cells were grown and treated with hydrogen peroxide as in the section above. A stock solution of Indo-1 AM (2 mg/ml) was added to the cell culture medium to a final concentration of 2 µg/ml. After a 30 min incubation at 37°C the cells were suspended with Trypsin-EDTA, transferred to centrifuge tubes and centrifuged for 6 min at 180 × g, 21°C. They were then gently washed twice, and the suspension of cells was transferred into fluorimeter cuvettes for spectroscopic analysis. Fluorescence intensity measurements were made with an ISS PC1 photon counting spectro fluorometer (Champagne, IL). Cells were stirred gently to avoid damage and to prevent them from settling. The excitation fluorescence was 346 nm and the emission fluorescence intensity was measured at 400 nm and 475 nm. Fluorescence intensities were corrected for the baseline. The fluorescence intensity ratio I_400_/I_475_ was used to estimate changes in ionized calcium concentration.

## RESULTS

**Fig. (1).**
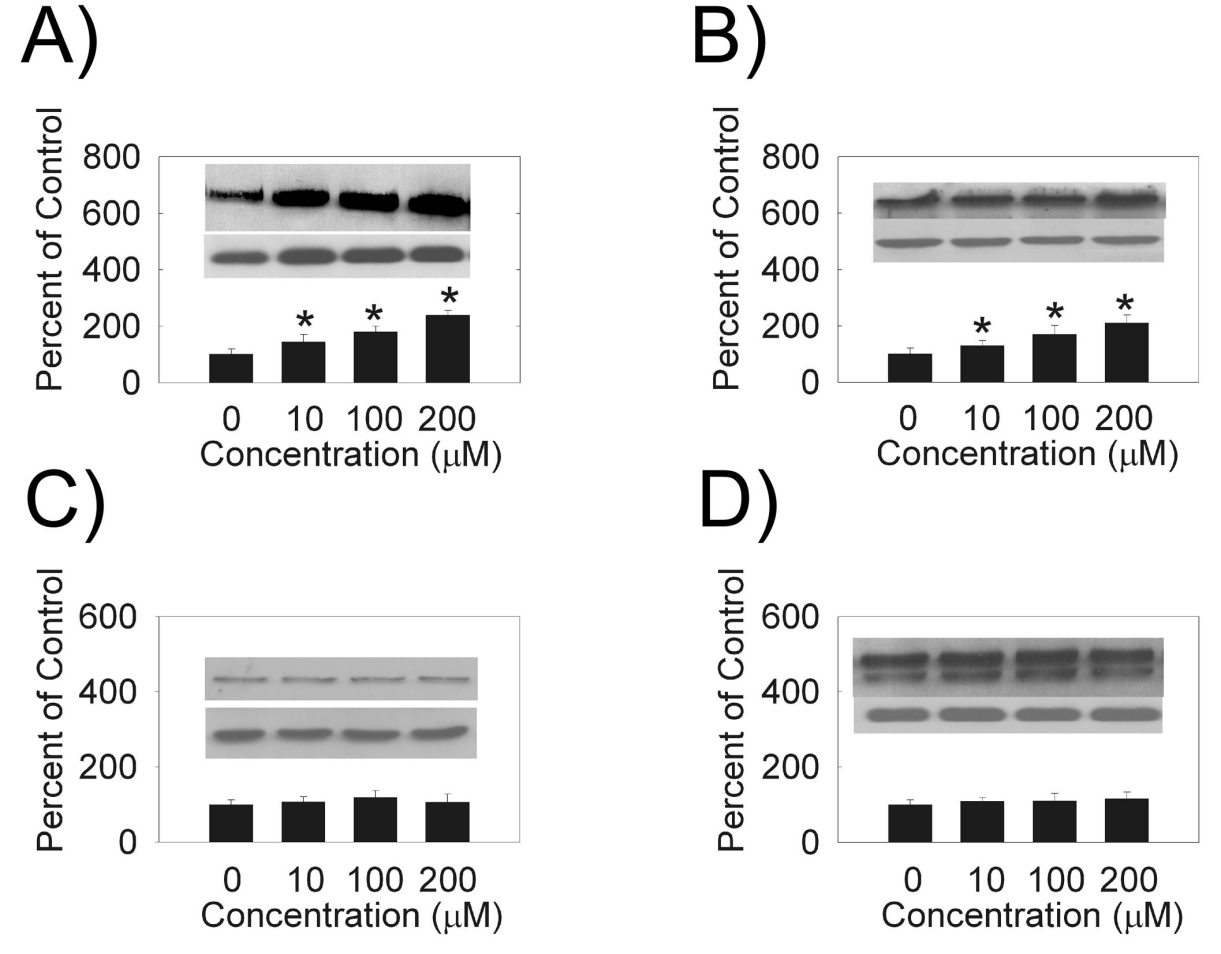
*Dose-dependence of hydrogen peroxide PMCA protein levels.* (**A**) PMCA1, 130 kDa (**B**) PMCA2, 120 kDa (**C**) PMCA3, 134 kDa (**D**) PMCA4 129 and 133 kDa. (Top Inset) A typical Western Blot showing dose-dependent effects of hydrogen peroxide on PMCA protein expression in the HLE B-3 cells treated for 4 hours. An equal amount of membrane protein was loaded in each lane and subjected to electrophoresis. Blots were incubated with a specific PMCA antibody, then stripped and reprobed with β-actin antibody (lower Western Blot insert, 42 kDa). (Bars) Densitometric analysis of Western Blots. Results are presented as mean ± standard error of 3 or 4 separate experiments. A value of P ≤ 0.05 was considered significant (*).

**Fig. (2).**
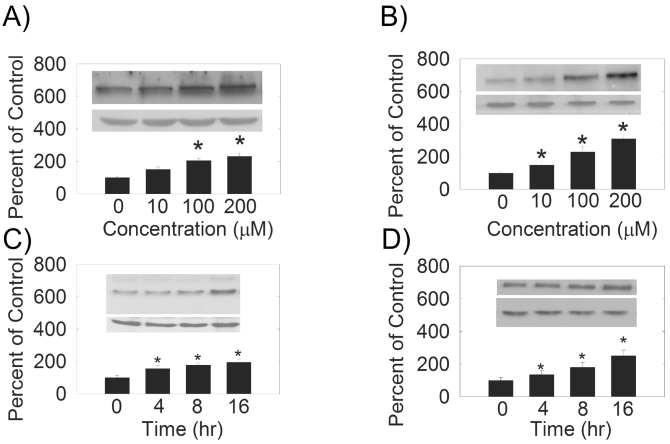
(**A**) and (**B**) Dose-dependence of hydrogen peroxide on SERCA protein levels. (**C**) and (**D**)  Hydrogen peroxide treatment time dependence of SERCA protein levels. (Top Inset) A typical Western Blot showing time-dependent effects of 10 µM hydrogen peroxide on SERCA protein expression in the HLE B-3 cells.  An equal amount of membrane protein was loaded in each lane and subjected to electrophoresis. A and C) SERCA2, 100-105 kDa, B and D) SERCA3, 97 kDa.  Blots were incubated with a specific SERCA antibody, then stripped and reprobed with β-actin antibody antibody (lower Western Blot insert, 42 kDa).  (Bars)  Densitometric analysis of Western Blots.

We examined the effects of hydrogen peroxide on the expression of PMCA and SERCA isoforms in HLE B-3 cells. We observed an increase in PMCA1 (Fig. **1A**), PMCA2 (Fig. **1B**), SERCA2 (Fig. **2A**) and SERCA3 (Fig. **2B**) levels with increasing hydrogen peroxide concentration. Quantification of PMCA1, PMCA2, SERCA2 and SERCA3 mRNA by quantitative real time RT-PCR (Table **1**) also showed a does-dependent increase in the mRNA levels. There were no differences between the expression of protein, or mRNA, for PMCA3 or PMCA4 (Figs. **1C**,**D**, Table **1**).

**Fig. (3).**
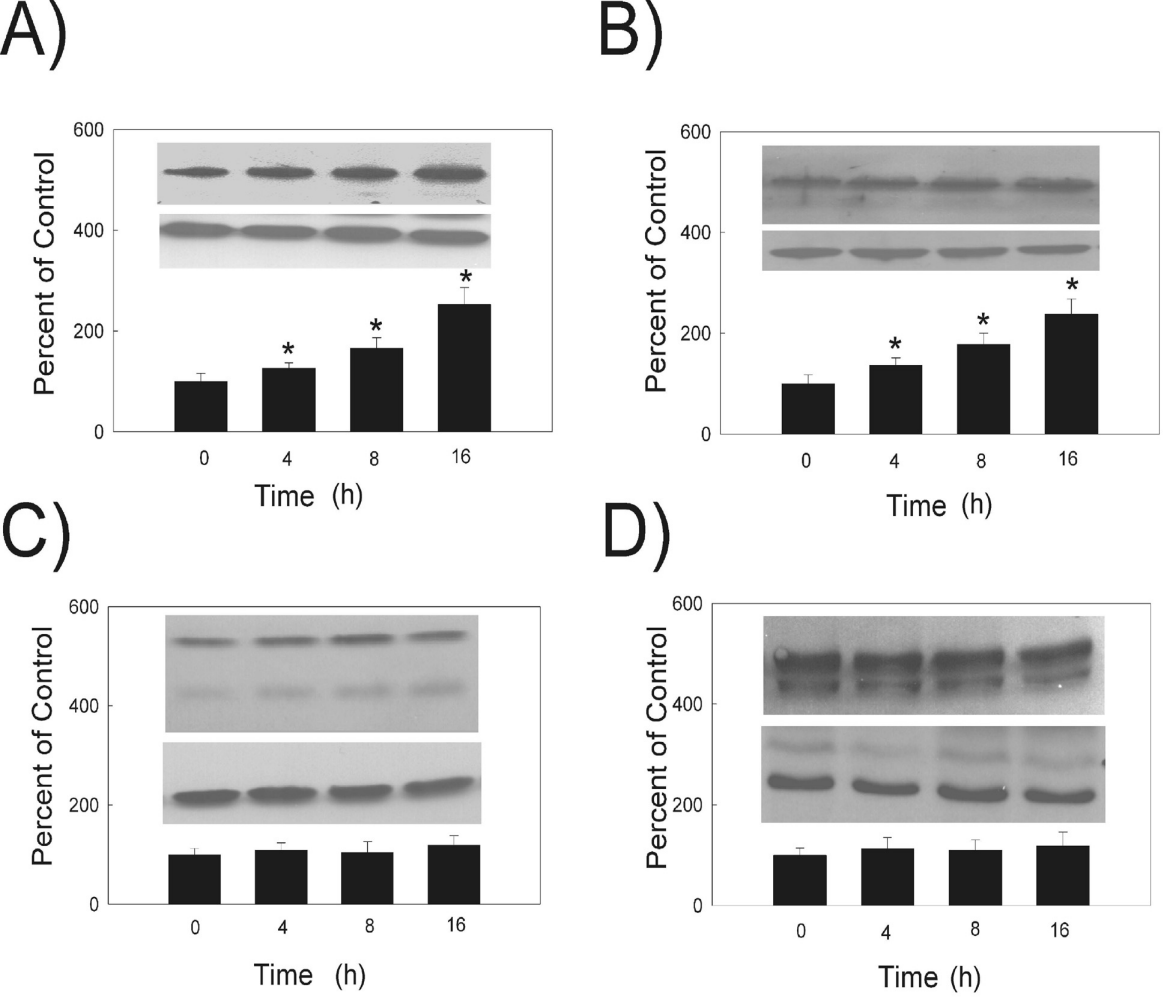
Hydrogen peroxide treatment time dependence of PMCA protein levels. (Top Inset) A typical Western Blot showing time-dependent effects of 10 µM hydrogen peroxide on PMCA protein expression in the HLE B-3 cells. (**A**) PMCA1, 130 kDa (**B**) PMCA2, 120 kDa (**C**) PMCA3, 134 kDa (**D**) PMCA4 129 and 133 kDa. An equal amount of membrane protein was loaded in each lane and subjected to electrophoresis. Blots were incubated with a specific PMCA antibody, then stripped and reprobed with β-actin antibody antibody (lower Western Blot insert, 42 kDa). (Bars) Densitometric analysis of Western Blots. Results are presented as mean ± standard error of 3 or 4 separate experiments. A value of P ≤ 0.05 was considered significant (*).

In a similar study, we evaluated the effects of hydrogen peroxide on the PMCA and SERCA protein and mRNA levels at different treatment times by both Western blot (Figs. **2C**,**D** and Fig. **3**) and quantitative real time RT-PCR techniques (Table **1**). Densitometric analysis of the protein bands showed that PMCA1 (Fig. **3A**), PMCA2 (Fig. **3B**), SERCA2 (Fig. **2C**) and SERCA3 (Fig. **2D**) protein levels were significantly increased with 10 μM hydrogen peroxide treatment in a time- dependent manner. The quantification of the level of mRNA by real time RT-PCR showed that after 4, 8, and 16 hours of hydrogen peroxide treatment, the levels of PMCA1, PMCA2, SERCA2 and SERCA3 mRNA increased significantly (Table **1**). The levels of PMCA3 and PMCA4 protein and mRNA did not change with treatment time (Table **1**).

**Fig. (4).**
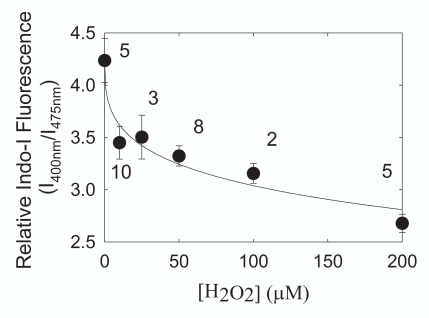
HLE B-3 cells were treated with hydrogen peroxide for 4 hours and the ratio of the fluorescence intensity of the calcium probe Indo-1 AM was used to measure changes in ionized intracellular calcium. A drop in the level of I400/I475 indicates hydrogen peroxide treatment caused intracellular calcium to rise. We estimate that calcium rose from 120 nM to 160 nM with 200 µM H2O2 treatment. Data are presented as mean ± standard error. Numbers next to the data points are the number of separate experiments

Cell calcium increased after 4 hours of hydrogen peroxide treatment (Fig. **4**). The increase in cell calcium with hydrogen peroxide was concentration dependent and could be fit (Sigma Plot 8.0, SPSS, Inc., Chicago IL) to a four-parameter logistic equation used to measure ligand binding (r^2^ = 0.944).

## DISCUSSION

To evaluate the effects of oxidation on lens epithelial cell PMCA and SERCA expression, we used a cell line derived from human lens epithelial cells, HLE B-3. In this study, we have shown that hydrogen peroxide treatment of HLE B-3 cells upregulates PMCA1, PMCA2 and SERCA2 and SERCA3 in a dose- and time-dependent manner while the PMCA3 and PMCA4 expression remained unchanged. In the present study, we treated the HLE B-3 cells with hydrogen peroxide to evaluate the responses of these cells to oxidative stress. The concentration of hydrogen peroxide used in these studies was in the range 10-200 μM which is only slightly higher than the hydrogen peroxide concentrations found in human cataract 1-75 μM [27,28]. Higher concentrations of hydrogen peroxide (> 200 μM) caused a fraction of cells to die. Wang *et al*. [40] found that 50 % of the HLE B-3 cells were not viable after 8 hours of treatment with 100 μM hydrogen peroxide due to the loss of mitochondrial function. We did not use “conditioned cells” that have been generated by gradually exposing the cells to higher concentrations of peroxides which causes the cells to survive in higher concentrations of peroxides compared to control cells [29]. These cells develop a complex antioxidant defense system which is comprised of high concentrations of catalase, gluthation-S-transferase and regulators of metal ion concentration, such as ferritin and hephaestin.

In our study, β-actin was used as an internal control for protein expression and GAPDH was used as an internal control for mRNA. The expression of β-actin may be influenced by H_2_O_2_ treatment [41]. Because the results for both mRNA and protein expression were consistent, the possibility that the controls were influenced by H_2_O_2_ is not likely.

Why do human lens epithelial cells have such a profound response to the oxidative stress which is manifested by a significant increase in the expression of calcium regulatory proteins, PMCA1, PMCA2, SERCA2 and SERCA3? The normal electrolyte composition of the healthy lens is achieved by the balance between passive ion leakage and active transport. Both aspects of ion regulation are compromised when the lens is exposed to hydrogen peroxide [42]. Electrophysiological experiments have shown that shortly after exposing the lens to hydrogen peroxide, there was a significant increase in the lens passive permeability and a partial impairment of the lens Na/K- pump [42]. The sensitivity of the lens transport pumps to the concentration of hydrogen peroxide changes abruptly around 100 μM. Lower concentrations have less effect on the ion transport pumps and lens ion content [42] probably because, high concentration of hydrogen peroxide (> 60 μM) overwhelm the antioxidant protection system of the lens [43].

The sum of lens SERCA and PMCA activity is substantially inhibited by μM levels of hydrogen peroxide [18,44] and in light of our findings, we speculate that hydrogen peroxide inhibits the calcium pumps in HLE B-3 cells. After treating HLE B-3 cells for 3 hours with 125 μM hydrogen peroxide, calcium influx doubled as a result of Ca^2+^-ATPase inhibition or increased membrane permeability [45]. PMCA1 was one of many proteins upregulated in HLE B-3 cells that were conditioned to withstand high levels of peroxide [46] in agreement with the present study. The fact that PMCA1 and SERCA3 are upregulated by hydrogen peroxide treatment as well as by increased intracellular calcium levels after thapsigargin treatment [21] or hydrogen peroxide treatment [45] indicates a concerted effort by the cells to overcome a deleterious peroxide-induced increase in calcium. Thus, oxidation-induced elevated calcium levels may be an initiating factor in the concerted upregulation of PMCA1 and SERCA3 [22].

The fact that PMCA2 was upregulated in human cataractous lenses [36] and in hydrogen peroxide treated HLE B-3 cells in this study, but not by thapsigargin [21], a plant derived natural compound that inhibits SERCA and elevates intracellular calcium, points to regulatory factors for PMCA2 other than calcium. Similarly, SERCA2 is upregulated with H_2_O_2_ treatment, but not up regulated by a thapsigargin induced increase in intracellular calcium. What the regulatory factors regulate are and whether other stresses regulate calcium pump expression remains to be determined. In previous studies [8,21,22] we made the assumption that we were measuring the protein expression of SERCA2b since this alternatively spliced isoform is expressed in all cell types and SERCA2a has been found only in muscle tissue. Until this assumption is tested with antibodies specific for SERCA2a and SERCA2b, we refrained from making the distinction between the alternatively spliced isoforms, a and b, of SERCA2. The 120bp nucleotides sequence that we used for quantitative real time PCR is specific for SERCA2b mRNA [22] and the partial sequence of the 270 bp fragment from SERCA2b PCR product is 100% homologus to the sequence encoded region from 2864-2983 bp of the human SERCA2b gene (M23115) [22]. In this study, mRNA specific for SERCA2b and protein expression for SERCA2 follow similar patterns of expression.

Why PMCA1 and PMCA2 are upregulated by hydrogen peroxide treatment and PMCA3 and PMCA4 are not is a difficult question to answer. PMCA isoform diversity, function and distribution have been reviewed [47]. As pointed out in the review [47], determining the function of individual isoforms is extremely difficult because usually more than one isoform is expressed in a tissue and there are no inhibitors specific for any of the PMCA isoforms or splice variants. Compared to PMCA4, PMCA2 is active at lower calcium, ATP and calmodulin concentrations [47-49], so in an oxidatively stressed cell with a low ATP concentration, expression or PMCA would be advantageous over the expression of PMCA4. Another advantage of selectively upregulating PMCA2 rather than PMCA3 is that PMCA2 has a 50 fold higher affinity for calmodulin than PMCA3 which is virtually a calmodulin-independent pump [47,50]. A complication to these speculations is that we did not quantify the over 30 splice variants of the PMCA’s and alternative splicing can impact the function of a PMCA isoform. For instance compared to the b splice form, the a splice form exhibits lower calmodulin binding and activation, lower calcium affinity and lower protein kinase C phosphorylation [47,49].

## CONCLUSIONS

Under a wide range of treatment times and concentrations used in this study, H_2_O_2 _did not decrease the expression of any of the Ca^2+^-ATPase isoforms measured, and for some isoforms, the expression of Ca^2+^-ATPase increased. The upregulation of SERCA after 4 hours of H_2_O_2_ treatment indicates that Ca^2+^-ATPase activity measurements in human lenses need to be interpreted cautiously since human lenses are often collected 4-24 hours post mortem. Our data suggest that oxidative stress could contribute to the rise in the level of PMCA2 expression in human cataractous lenses compared with age matched clear lenses [36].

## Figures and Tables

**Table 1.** PMCA and SERCA mRNA Expression Versus Treatment Time and H_2_O_2_ Concentration

	4 hr, 10 µM	8 hr, 10 µM	16 hr, 10 µM	4 hr, 10 µM	4 hr, 100 µM	4 hr, 200 µM
**PMCA1 mRNA**	1.8 ± 0.2	2.2 ± 0.3	3.3 ± 0.3	1.8 ± 0.3	2.4 ± 0.2	3.0 ± 0.3
**PMCA2 mRNA**	1.5 ± 0.2	2.0 ± 0.3	2.5 ± 0.2	1.6 ± 0.1	2.0 ± 0.2	2.9 ± 0.3
**PMCA3 mRNA**	1.1 ± 0.2	1.2 ± 0.2	1.2 ± 0.1	1.2 ± 0.2	1.2 ± 0.2	1.3 ± 0.3
**PMCA4 mRNA**	1.1 ± 0.2	1.1 ± 0.2	1.2 ± 0.2	1.1 ± 0.2	1.1 ± 0.2	1.2 ± 0.2
**SERCA2 mRNA**	1.5 ± 0.2	2.1 ± 0.2	2.9 ± 0.3	1.9 ± 0.2	2.4 ± 0.3	3.4 ± 0.3
**SERCA3 mRNA**	1.6 ± 0.3	2.0 ± 0.2	3.1 ± 0.3	1.8 ± 0.3	3.0 ± 0.3	3.4 ± 0.4

PMCA1 and 2 and SERCA 2 and 3 are statistically different, p < 0.05.

Data are average ± standard error of the mean, n=3-4.
